# Increased cold injuries and the effect of body mass index in patients with peripheral vascular disease

**DOI:** 10.1186/s12889-020-09789-w

**Published:** 2021-02-05

**Authors:** Jin-young Min, Yeon-Soo Choi, Hyeong-Seong Lee, Sohyae Lee, Kyoung-bok Min

**Affiliations:** 1 Veterans Medical Research Institute, Veterans Health Service Medical Center, Seoul, Republic of Korea; 2 Department of Statistics and Data Science, College of Commerce and Economics, Yonsei University, Seoul, Republic of Korea; 3 Department of Preventive Medicine, College of Medicine, Seoul National University, 103 Daehak-ro, Jongno-gu, Seoul Seoul, 110-799 Republic of Korea

**Keywords:** Non-freezing cold injury, Winter, Peripheral vascular disease, Body fat, General population

## Abstract

**Background:**

Exposure to extremely or moderate low temperatures is associated with increased morbidity and mortality risk. Peripheral vascular disease (PVD) is a slow and progressive circulation disorder. Given that cold temperature causes constriction of the small arteries and veins in the skin, patients who suffer from peripheral circulation problems, like PVD, may be vulnerable to cold injuries. This study aimed to investigate the association between PVD and cold-induced injuries in the winter among Korean adults. We further analyzed the association stratified by body mass index (BMI) classification.

**Methods:**

We used the 2002–2015 National Health Insurance Service-National Sample Cohort data and included a total of 535,186 adults as the study population. Patients with underlying PVD were identified by ICD-10 code I73. Cold-related illnesses were defined by ICD-10 codes (T690, T691, T698, T699, T330 ~ T339, T340 ~ T349, and T350 ~ T357). Body mass index (BMI) was categorized into underweight, normal weight, overweight, and obese.

**Results:**

A total of 23.21% (*n* = 124,224) were PVD patients, and 0.59% (*n* = 3154) had cold-induced injuries. PVD patients were more likely to be diagnosed with cold injuries, but it was valid only in the underweight or normal weight groups. After adjusting for age, sex, income, cigarette smoking, alcohol consumption, regular exercise, high blood pressure, and hyperglycemia, PVD patients had a significantly increased odds ratio (OR) for cold injuries [adjusted OR = 1.11; 95% confidence intervals (95% CI): 1.01–1.21]. Increased OR for cold injuries in PVD patients was also observed in adults (adjusted OR = 1.14; 95% CI: 1.03–1.25 in Model 2), but not in the elderly. When we classified study subjects into the four BMI groups, the adjusted OR of cold injuries in PVD patients was significant in the underweight group (OR = 1.83; 95% CI, 1.26–2.66) and normal weight group (OR = 1.15; 95% CI, 1.03–1.27), not in those with overweight and obese. In adults, a consistent result was found in adults in the underweight group (OR = 1.63; 95% CI, 1.08–2.47 in Model 2) and normal weight group (OR = 1.19; 95% CI, 1.07–1.33 in Model 2). In the elderly, the adjusted OR for cold injuries was only significant in the underweight group (OR = 3.37; 95% CI, 1.08–10.53 in Model 2).

**Conclusions:**

We found a significant association between PVD and cold-induced injuries in the general population. BMI modified the association. Thus, the association observed appears to be clinically applicable to PVD patients being low to normal BMI.

## Background

Temperature is a crucial factor in health and diseases. Coupled with climate change issues expected to increase the frequency and strength of extreme weather events, interest in the health effect of temperature extremes have increased [1]. Many epidemiologic studies have reported that moderate-to-extremely low temperature is associated with increased rates of hospital visits, admission, and death due to cardiac, cerebrovascular and respiratory diseases, among other causes [2, 3].

Less commonly investigated are cold-induced injuries, including freezing and non-freezing injuries. When the body is exposed to cold, sympathetically-mediated vasoconstriction is elicited to reduce blood flow to the peripheries [4–6]. This vasoconstriction response maintains the body core temperature and decrease peripheral tissue temperatures [4–6]. Continued cold exposure and vasoconstriction can contribute to peripheral cold injuries [4–6]. The primary concerns related to cold injury risks are the military, athletes, and workers who are experiencing the dangers of severely cold environments [7–11]. In an era of climate change wherein extreme weather events are becoming increasingly common, the risk of cold injury applies to these specific high-risk individuals and the general population [9, 10, 12]. A better understanding of factors that affect cold-induced injuries in the general population is needed. This information would be useful for targeting vulnerable people and mediating the effects of cold weather.

Peripheral vascular disease (PVD) is a gradually progressive circulation disorder [13, 14]. PVD results from thickening of the lining of blood vessels caused by a buildup of plaque and leads to narrowing, blockage, or spasms in the arterial and venous blood vessels [13, 14]. It can reduce blood circulation to the specific organs or body regions supplied by these vessels. The legs and feet are most often affected, but upper extremity involvement is not uncommon [13]. Given that cold exposure causes constriction of the small arteries and veins in the skin [4, 6], patients who experience peripheral circulation issues may be vulnerable to cold injuries.

We aimed to investigate the association between PVD and cold-induced injuries during the winter season in Korean adults. Based on the evidence that a higher body fat percentage increases insulation [15], we further analyzed the association stratified by body mass index (BMI).

## Methods

### Data source and study population

The study population comprised the 2002–2012 National Health Insurance Service-National Sample Cohort (NHIS-NSC). Details on the generation and overview of the data have been described in previous papers [16]. Briefly, South Korea has an NHI system, which provides mandatory social health insurance for the entire population to achieve universal health care coverage. The NHI employs paid health care providers on a fee-for-service basis and contains all data necessary for reimbursement in terms of patients’ demographics, medical diagnosis, procedures, treatment, prescription medications, and insurers’ payment coverage. The NHIS record is valuable data to make the system’s effectiveness and relevance to public health and medical research.

The NHIS-NSC aims to provide public health scientists and policymakers with representative, useful information regarding citizens’ utilization of health insurance and health examinations. A representative sample cohort was selected from the target population through systematic stratified random sampling in each stratum, which is proportional to an individual’s total annual medical expenses, including age, sex, residence, and health insurance type as target variables for sampling [16]. To ensure the representativeness of the NHIS-NSC sample, whether a 95% confidence interval of the sample’s average total annual medical expenses included the population average was evaluated. It was satisfied for all strata [16]. A total of 1,103,405 sample subjects, which corresponds to approximately 2.2% of the total Korean population, were included in the dataset.

In the current study, we initially included 721,630 adults aged more than 20 years. Of them, 186,444 subjects who had incomplete medical records or other variables of interest were excluded; most of these variables were related to subjects’ health behaviors. Thus, the final study sample comprised of 535,186 adults.

### Definition of PVD and cold injuries

The definition of PVD was based on the tenth revision of the International Statistical Classification of Diseases and Related Health Problems (ICD-10). Patients with underlying PVD were defined as people diagnosed with I73 of the ICD-10 before being diagnosed with any cold-related illnesses.

The main outcome variable was cold-induced injuries. Cold injuries are divided into systematic hypothermia and localized injuries. Hypothermia is a medical emergency and is not discussed in detail here. This study focused only on localized injuries such as freezing and non-freezing injuries.

Cold injury was also defined according to the ICD-10. The specific diagnostic codes were T69.0 (Immersion hand and foot), T69.1 (Chilblains), T69.8 (Other specified effects of reduced temperature), T69.9 (Effect of reduced temperature, unspecified), T33.0–9 (Superficial frostbite), T34.0–9 (Frostbite with tissue necrosis), and T35.0–7 (Unspecified frostbite).

### Variables of interest

BMI was calculated as weight in kg divided by height in m^2^. BMI was used to define subjects as underweight (BMI of < 18.50 kg/m^2^), normal weight (BMI of 18.50–24.99 kg/m^2^), overweight (BMI of 25.00–29.99 kg/m^2^), or obese (BMI of ≥30.00 kg/m^2^).

The other variables were confounding factors that influenced both the dependent and independent variables, causing a spurious association. From the literature [17–19], subject characteristics, such as demographics, health behavior, and clinical health conditions, were included as confounding variables. Specifically, the demographic variables were age (grouped by decade: 20–29, 30–39, 40–49, 50–59, or 60–64 years), sex (male or female), and household income (quartiles as < 25, 25–50%, 50–75%, and > 75%). The health behavior variables were cigarette smoking (current smoker, former smoker, or never smoker), current alcohol consumption (yes or no), and regular exercise status (yes or no). Clinical condition variables were high blood pressure (systolic blood pressure of > 140 mm/Hg or diastolic blood pressure of > 90 mm/Hg) and hyperglycemia (blood glucose of > 125 mg/dL).

### Statistical analyses

Participants’ characteristics are presented as numbers and percentages, in which the differences in those with and without cold injuries were tested by the Chi-squared test. Logistic regression analysis was conducted to determine the association between PVD and cold-induced injuries by setting patients without PVD as a reference. Odds ratio (OR) and 95% confidence interval (CI) were calculated for the outcome variable according to the presence of PVD. To examine the effect of body fat on the concerned association, we further analyzed the logistic regression according to BMI classification - underweight (BMI of < 18.50 kg/m^2^), normal weight (BMI of 18.50–24.99 kg/m^2^), overweight (BMI of 25.00–29.99 kg/m^2^), or obese (BMI of ≥30.00 kg/m^2^). All regression models were adjusted for confounding variables, including age, sex, income, cigarette smoking, alcohol consumption, regular exercise, high blood pressure, and hyperglycemia. Since age is known to be a crucial risk factor for PVD and thermoregulation [1, 13, 14], we performed post-hoc subgroup analyses by age - adults (20 ~ 64 years) and elderly (≥65 years). The results from subgroups help ensure the robustness of the association between PVD and cold injuries. Statistical analyses were performed using SAS 9.4 software, and *P*-values of ≤0.05 were considered to indicate statistical significance.

## Results

**Fig. 1:**
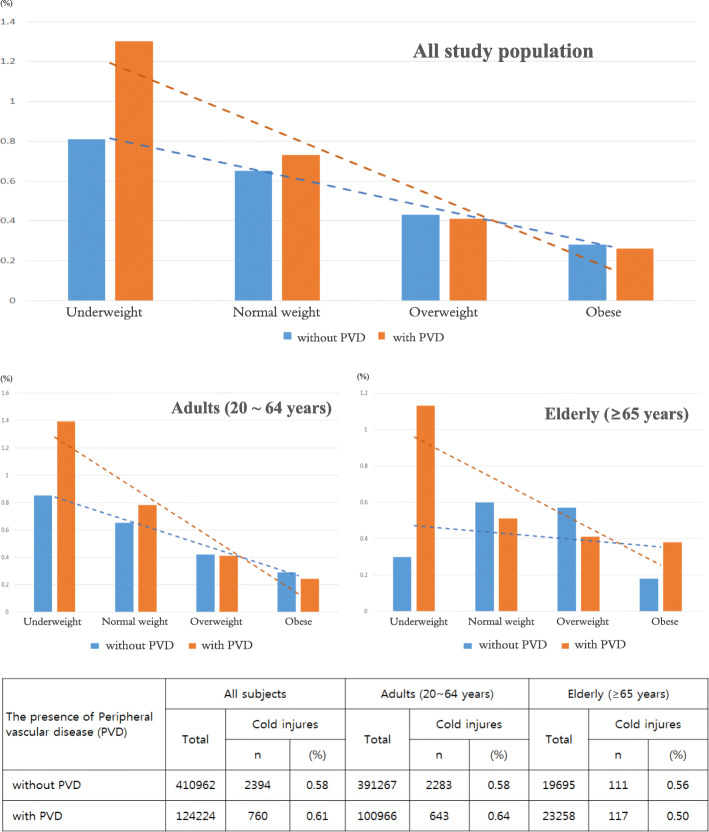
Percentages of cold injured subjects by the presence of PVD and BMI classification, stratified by age. BMI was classified as ‘underweight (< 18.5 kg/m^2^)’, ‘normal weight (18.5-24.9kg/m^2^)’, ‘overweight (25–29.9 kg/m^2^)’, and ‘obese (≥30 kg/m^2^)’

Figure 1 shows the percentage of cold-injured subjects by the presence of PVD and BMI classification. A total of 124,224 (23.21%) were PVD patients, which included 100,966 adults aged 20 ~ 64 years (20.51%) and 23,258 elderly aged ≥65 years (54.15%). The prevalence of cold injuries was slightly higher in PVD patients than in non-PVD patients (0.61% vs. 0.58%). In adults, PVD patients were more likely to have clod injures than non-PVD patients (0.64% vs. 0.58%), but, in the elderly, the prevalence of cold injuries was higher in non-PVD patients than in PVD patients (0.56% vs. 0.50%). Overall, the highest prevalence of cold injuries was observed among underweight subjects, and the lowest prevalence was among obese subjects. We stratified subjects according to the presence of PVD and BMI classification. We observed that underweight patients with PVD were the most likely to experience cold injuries, and those who were obese with PVD were less likely to experience cold injuries. A similar trend was observed in both adults (20 ~ 64 years) and the elderly (≥65 years).

Table 1 presents the characteristics of study population by the presence of cold-induced injuries. Of 535,186 subjects, 3154 (0.59%) were had cold-induced injuries. In the demographic distribution, cold injured subjects were more likely to be young adults aged 20–29 years, to be female, and to have the lowest household income (≤25%) than their counterparts. Further, cold injured subjects were more likely to be non-smokers and non-drinkers and to not engage in regular exercise. Finally, cold injured subjects were more likely to be underweight or normal weight and had no high blood pressure or high blood glucose levels than were those without cold injuries.

**Table 1:** Subjects’ characteristics by the presence of cold injuries unit: n (%)

	With cold injuries (n = 3154)	Without cold injuries (*n* = 532,032)	*p*-value^a^
Age (year)			
20–29	727 (23.05)	110,472 (20.76)	0.0098
30–39	761 (24.13)	135,853 (25.53)	
40–49	790 (25.05)	133,390 (25.07)	
50–59	463 (14.68)	79,013 (14.85)	
60–69	320 (10.15)	53,174 (9.99)	
70–79	84 (2.66)	17,557 (3.30)	
80-	9 (0.29)	2573 (0.48)	
Sex			
Male	1289 (40.87)	268,627 (50.49)	< 0.0001
Female	1865 (59.13)	263,405 (49.51)	
Household income			
≤ 25%	1072 (33.99)	162,062 (30.46)	< 0.0001
26–50%	678 (21.50)	117,951 (22.17)	
51–74%	708 (22.45)	121,472 (22.83)	
≥ 75%	696 (22.07)	130,547 (24.54)	
Smoking status			
Current smoker	622 (19.72)	144,901 (27.24)	< 0.0001
Past smoker	257 (8.15)	48,550 (9.13)	
Never smoker	2275 (72.13)	338,581 (63.64)	
Alcohol drinking			
No	1784 (56.56)	277,416 (52.14)	< 0.0001
Yes	1370 (43.44)	254,616 (47.86)	
Regular exercise			
No	1868 (59.23)	298,930 (56.19)	0.0006
Yes	1286 (40.77)	233,102 (43.81)	
BMI classification^b^			
Underweight	196 (6.21)	21,888 (4.11)	< 0.0001
Normal weight	2262 (71.72)	339,002 (63.72)	
Overweight	642 (20.36)	151,634 (28.50)	
Obese	54 (1.71)	19,508 (3.67)	
High blood pressure^c^			
No	2874 (91.12)	463,475 (87.11)	< 0.0001
Yes	280 (8.88)	68,557 (12.89)	
Hyperglycemia^d^			
No	3004 (95.24)	498,660 (93.73)	0.0005
Yes	150 (4.76)	33,372 (6.27)	

^a^p-value was calculated using the Chi-square test for categorical variables

^b^BMI was categorized by underweight (< 18.5 kg/m^2^), normal weight (18.5–24.9 kg/m^2^), overweight (25–29.9 kg/m^2^), and obese (≥30 kg/m^2^)

^c^High blood pressure was indicated if systolic/diastolic was over 140/90 (mm/Hg)

^d^Hyperglycemia was indicated if blood glucose was greater than 125(mg/dl)

Table 2 indicates the OR of cold-induced injuries by subjects’ characteristics and BMI classification. Age categories showed no difference in the OR for cold injuries, but female subjects were more likely to have cold injuries than male subjects. Among underweight subjects, current or past smokers were less frequently diagnosed with cold injuries than never smokers (*p* = 0.0129). The OR of cold injuries in normal weight subjects varied depending on household income (the lowest OR in the low income group, *p* = 0.0072), smoking (the lowest OR in past smokers, *p* < 0.0001), drinking (the lowest OR in current drinkers, *p* = 0.0001), exercise (the lowest OR in those engaging in regular exercise, *p* = 0.0053), and high blood pressure (the lowest OR in those with hypertension, *p* = 0.0003). Among overweight subjects, current smokers, alcohol drinkers, and hypertensive subjects were less frequently diagnosed with cold injuries than non-smokers (*p* = 0.0006), non-alcohol drinkers (*p* = 0.0189), and non-hypertensive subjects (*p* = 0.0009), respectively. Among obese subjects, the OR for cold injuries was lower in alcohol drinkers than in non-alcohol drinkers (*p* = 0.0215).

**Table 2:** OR (95% CI) of cold injuries by subject’s characteristics, stratified by BMI classification

Characteristics	Underweight (< 18.5 kg/m^2^)			Normal weight (18.5–24.9 kg/m^2^)			Overweight (25–29.9 kg/m^2^)			Obese (≥30 kg/m^2^)		
	OR	(95% CI)	*p*-value	OR	(95% CI)	*p*-value	OR	(95% CI)	*p*-value	OR	(95% CI)	*p*-value
Age (year)			0.2260			0.0914			0.1297			0.9767
20–29	Reference			Reference			Reference			Reference		
30–39	0.84	(0.58–1.21)		0.92	(0.82–1.04)		0.79	(0.61–1.04)		1.11	(0.51–2.42)	
40–49	0.80	(0.49–1.31)		0.97	(0.87–1.10)		0.98	(0.76–1.26)		0.88	(0.37–2.07)	
50–59	1.38	(0.84–2.27)		0.91	(0.79–1.05)		1.11	(0.85–1.44)		1.30	(0.55–3.07)	
60–69	1.08	(0.64–1.82)		0.94	(0.80–1.11)		1.12	(0.84–1.50)		1.34	(0.52–3.46)	
70–79	0.42	(0.17–1.03)		0.74	(0.57–0.97)		0.97	(0.60–1.57)		0.86	(0.11–6.68)	
80-	0.58	(0.14–2.35)		0.33	(0.12–0.87)		1.59	(0.50–5.05)		NC	(NC-NC)	
Sex			0.0283			< 0.0001			0.0125			0.0180
vMale	Reference			Reference			Reference			Reference		
Female	1.44	(1.04–2.00)		1.46	(1.34–1.59)		1.22	(1.04–1.42)		1.98	(1.13–3.49)	
Household income			0.7225									
≤ 25%	Reference			Reference		0.0072	Reference		0.0852	Reference		0.3805
26–50%	0.88	(0.60–1.29)		0.90	(0.80–1.00)		0.79	(0.63–0.98)		1.01	(0.52–1.95)	
51–74%	1.08	(0.74–1.57)		0.90	(0.81–1.01)		0.82	(0.67–1.02)		0.69	(0.32–1.46)	
≥ 75%	0.88	(0.59–1.31)		0.82	(0.73–0.92)		0.81	(0.66–1.00)		0.54	(0.24–1.22)	
Smoking status			0.0129			< 0.0001			0.0006			0.1156
Never smoker	Reference			Reference			Reference			Reference		
Current smoker	0.65	(0.45–0.95)		0.64	(0.58–0.71)		0.69	(0.57–0.84)		0.61	(0.31–1.18)	
Past smoker	0.35	(0.13–0.94)		0.81	(0.69–0.95)		0.99	(0.78–1.25)		0.31	(0.08–1.30)	
Alcohol drinking			0.8116			0.0001			0.0189			0.0215
No	Reference			Reference			Reference			Reference		
Yes	0.97	(0.73–1.29)		0.85	(0.78–0.92)		0.83	(0.71–0.97)		0.50	(0.28–0.90)	
Regular exercise			0.3623			0.0051			0.7721			0.7864
No	Reference			Reference			Reference			Reference		
Yes	0.86	(0.63–1.19)		0.89	(0.82–0.96)		0.98	(0.84–1.14)		0.93	(0.54–1.59)	
High blood pressure^c^			0.1973			0.0003			0.0009			0.8034
No	Reference			Reference			Reference			Reference		
Yes	0.59	(0.26–1.32)		0.75	(0.64–0.88)		0.68	(0.55–0.86)		0.93	(0.50–1.70)	
Hyperglycemia^d^			0.4259			0.1175			0.1310		0.2632	
No	Reference			Reference			Reference			Reference		
Yes	0.67	(0.25–1.80)		0.85	(0.69–1.04)		0.79	(0.59–1.07)		0.56	(0.20–1.55)	

^a^p-value was calculated using the Chi-square test for categorical variables

^b^High blood pressure was indicated if systolic/diastolic was over 140/90 (mm/Hg)

^c^Hyperglycemia was indicated if blood sugar was greater than 125(mg/dl)

Table 3 shows the OR (95% CI) for the diagnosis of cold injuries according to the presence of PVD and BMI classification. Model 1 was adjusted for sociodemographic variables (i.e., age, sex, and household income). Model 2 was further adjusted for cigarette smoking, alcohol consumption, regular exercise, high blood pressure, and hyperglycemia. When we set non-PVD patients as a reference, PVD patients had a significantly higher OR for cold injuries (adjusted OR = 1.11; 95% CI: 1.01–1.21 in Model 2). Increased OR for cold injuries in PVD patients was also observed in adults (adjusted OR = 1.14; 95% CI: 1.03–1.25 in Model 2), but not in the elderly. The association between PVD and cold-induced injures was further examined according to BMI classification. Among the total population, PVD patients’ odds of being diagnosed with cold injuries were decreased with increasing BMI. The adjusted OR of cold injuries in PVD patients was significant in the underweight group (OR = 1.83; 95% CI, 1.26–2.66 in Model 2) and normal weight group (OR = 1.15; 95% CI, 1.03–1.27 in Model 2), but not in the overweight and obese groups. A consistent result was found in adults in the underweight group (OR = 1.63; 95% CI, 1.08–2.47 in Model 2) and normal weight group (OR = 1.19; 95% CI, 1.07–1.33 in Model 2). In the elderly, the adjusted OR for cold injuries was only significant in the underweight group (OR = 3.37; 95% CI, 1.08–10.53 in Model 2).

**Table 3:** OR (95% CI) of cold injures in PVD patients

	no. of case / all subjects	Unadjusted Model	Adjusted Model	
			Model 1	Model 2
Total population				
Overall	3154 / 535,186	1.05 (0.97–1.14)	1.04 (0.95–1.14)	1.11 (1.01–1.21)
BMI classification				
Underweight (< 18.5 kg/m^2^)	196 / 22,084	1.61 (1.15–2.26)	1.80 (1.24–2.61)	1.83 (1.26–2.66)
Normal weight (18.5–24.9 kg/m^2^)	2262 / 341,264	1.13 (1.02–1.24)	1.14 (1.02–1.26)	1.15 (1.03–1.27)
Overweight (25–29.9 kg/m^2^)	642 / 152,276	0.97 (0.81–1.15)	0.86 (0.72–1.04)	0.88 (0.73–1.06)
Obese (≥30 kg/m^2^)	54 / 19,562	0.92 (0.51–1.67)	0.76 (0.40–1.47)	0.77 (0.40–1.48)
Adults (20 ~ 64 years)				
Overall	2926 / 492,233	1.09 (1.00–1.19)	1.06 (0.97–1.17)	1.14 (1.03–1.25)
BMI classification				
Underweight (< 18.5 kg/m^2^)	179/19604	1.65 (1.11–2.43)	1.60 (1.06–2.42)	1.63 (1.08–2.47)
Normal weight (18.5–24.9 kg/m^2^)	2116/314803	1.21 (1.09–1.34)	1.18 (1.06–1.32)	1.19 (1.07–1.33)
Overweight (25–29.9 kg/m^2^)	581/139601	0.98 (0.82–1.19)	0.89 (0.73–1.09)	0.91 (0.74–1.11)
Obese (≥30 kg/m^2^)	50/18225	0.84 (0.44–1.61)	0.69 (0.34–1.39)	0.69 (0.34–1.41)
Elderly (≥65 years)				
Overall	228 / 42,953	0.89 (0.69–1.16)	0.90 (0.70–1.18)	0.92 (0.71–1.20)
BMI classification				
Underweight (< 18.5 kg/m^2^)	17 / 2470	3.77(1.23–11.58)	3.72 (1.20–11.53)	3.37 (1.08–10.53)
Normal weight (18.5–24.9 kg/m^2^)	146 / 26,397	0.84(0.60–1.20)	0.85 (0.61–1.18)	0.85 (0.61–1.18)
Overweight (25–29.9 kg/m^2^)	61 / 12,658	0.72 (0.44–1.19)	0.72 (0.43–1.20)	0.73 (0.44–1.21)
Obese (≥30 kg/m^2^)	4 / 1336	2.05 (0.21–19.78)	2.05 (0.21–20.22)	2.34 (0.22–24.83)

Model 1 was adjusted for age, sex, and household income

Model 2 was further adjusted for cigarette smoking, alcohol consumption, regular exercise, high blood pressure and hyperglycemia

## Discussion

Exposure to cold poses significant health risks. One of the direct effects is cold-induced injuries, including frostbite and Chilblains. The present study provides evidence for a potential link between PVD and cold-induced injuries in the general population. Our results indicated that PVD was associated with 11% increased odds of cold injuries during the winter (November to February). The association was modified by the BMI. Underweight PVD patients had the highest odds of cold-injuries among all BMI categories. A similar trend was observed in adults and the elderly. This finding implies that PVD patients may experience an increased incidence of freezing or non-freezing cold injuries during the winter, to which underweight patients may be particularly susceptible.

Cold injuries include a central effect such as hypothermia and a localized effect such as frostbite [20]. Our study focuses on localized cold inquires occurring in the winter seasons and encompassing freezing and non-freezing cold injuries. Freezing cold injuries, such as frostbite and frost nip, are defined as cold damage to the extremities caused by freezing the skin and underlying tissues at temperatures below approximately − 0.55 °C [20, 21]. Non-freezing cold injuries, such as trench foot and chilblains, occur when tissue fluids are exposed to low temperatures close to freezing point (0–15 °C) but not involving tissue freezing [20, 21]. Previous studies on cold-induced injuries have been mainly conducted with the military, sportspeople, and industrial settings and demonstrated risk factors such as increasing age, Caucasian, smokers, male sex, and inadequate clothing identified [20–24]. Little is known of the epidemiology of cold injuries in the general population.

Both acute and prolonged cold exposures affect vascular responses and worse preexisting vascular diseases [25, 26]. Cold temperature can increase arterial pressure, peripheral resistance, and cardiac demand, thus increasing vascular-related morbidity and mortality [15, 25–27]. However, there is a lack of evidence to show a higher risk of cold injuries among patients with vascular disease. To the best of our knowledge, this is the first report to demonstrate that PVD may be a risk factor for cold-induced injuries. Although no studies exist for comparison, this conclusion is biologically plausible, considering cold-induced vasoconstriction, the hyper-responsiveness of the extremities, and body fat’s role as insulation.

Cutaneous vasoconstriction is an essential response to thermoregulation. During cold exposure, the cutaneous vasoconstriction reflex mediated by the sympathetic nervous system occurs as the “first line of defense” against an excess reduction in body temperature [4, 28]. Cutaneous vasoconstriction decreases blood flow to the skin and lowers the skin temperature, limiting heat loss to the environment [4, 28]. Sympathetic vasoconstrictor nerves in the skin are tonically active in a thermoneutral environment. Their activities are responsible for changes in blood flow to the skin caused by daily activities or environmental temperature, in the way of increasing skin blood flow during cold exposure [4].

Although cold-induced peripheral vasoconstriction is a normal physiological process required to maintain thermal homeostasis, the response seems to be counterproductive in some cases [4]. Raynaud’s disease is an example. Raynaud’s disease is a disorder characterized by episodic spasms of the small arteries – such as those in the fingers and toes – in response to cold or emotional stress [29]. Raynaud’s disease causes hyper-activation of the sympathetic nerves induced by vasoconstriction, leading to blood circulation damage and further peripheral ischemia [29]. Under this ischemia, ice crystals may form in-cell (in rapid freezing) or outside of cells (in gradual freezing) and modify cell proteins, lipids, electrolytes, and hydration [30]. This may lead to cell degradation and tissue necrosis [30]. When thawing and refreezing occur, tissue integrity is rapidly exacerbated by ischemia [30]. Given that Raynaud’s disease is a functional PVD related to constriction of the peripheral blood vessels, our results on the high odds of cold injuries in PVD patients may be understandable within this context.

PVD is a disorder of the circulatory system outside of the brain and heart and is the most common disease of the arteries [14]. When an artery becomes blocked or narrowed, blood flow restricts, and the oxygen supply to the extremities is not sufficient; this is known as ischemia [14]. Although ischemia in PVD occurs for different reasons for ischemia in Raynaud’s disease, as noted earlier, ischemia in PVD patients may also cause the formation of ice crystals or an increase in tissue damage. As a result, prolonged and repeated cold exposure may increase the susceptibility of PVD patients to freezing or non-freezing cold injuries. Our findings need to be reproduced, and the underlying biological explanation should be identified through further studies.

We also observed the effect of BMI on the link between PVD and cold injuries. When stratified by BMI – underweight, normal weight, overweight, and obese – the odds of cold injuries in PVD patients were significantly increased in underweight patients compared to those of normal weight. In contrast, no significant association was found in the overweight and obese groups. Herein, our concern is whether a lower BMI is associated with an increased risk of cold injuries. This is valuable information to identify individuals at high risk of cold injuries, but only a few studies suggest their association. Akkurt et al. (2014) compared the demographic and clinical characteristics of sixty-nine patients with chilblains [31]. The mean BMI of patients with chilblains was significantly lower than that of controls (20.5 kg/m^2^ vs. 22.4 kg/m^2^; *p* = 0.01). The authors suggested that a lower BMI indicates less adipose tissue and less protection against cold exposure; thus, a low BMI could contribute to the development of chilblains [31]. A US study reviewed medical records and death certificates during the cold season in New York to identify risk factors for cold-related illnesses and deaths [32]. The data showed that decedents were significantly more likely to be normal or underweight than the general population (58% vs. 43%, *p* ≤ 0.001). Stjernbrandt et al. (2014) conducted a nested case-control study to identify factors associated with cold sensitivity [33]. Regarding individual factors, being overweight was associated with a lower reported frequency of cold sensitivity (OR 0.5; 95% CI 0.4–0.7) than normal weight, suggesting that overweight acts as a protective factor, possibly through a passive insulating mechanism [33]. These results, including our observation, highlight the potential link between lower BMI and cold injuries; but, there is a conflicting result. Raza et al. (2010) compared the BMI of chilblains patients with controls and found no significant difference [34]. More research is needed to confirm a causal relationship between low BMI and an increased risk of cold injuries.

Previous studies have reported a positive association between BMI and body temperature [35, 36]. In cold environments, when vasoconstriction has occurred, a thicker layer of subcutaneous fat provides an extra layer of thickness and acts as insulation for maintaining body temperature [37, 38]. Castellani et al. (2006) reported that athletes with a higher body fat percentage had a more stable core temperature than those with a lower body fat percentage [15]. Older adults aged > 60 years and younger children with low subcutaneous fat were considered at risk of cold-induced injury [15]. Although the current data cannot provide the exact mechanism on the role of BMI in linking PVD and cold injuries, PVD patients with relatively less body fat (that is, underweight and normal weight patients) may be less tolerant of the cold injuries than those with more body fat (that is, overweight and obese), likely because of the reduced insulating properties of fat against heat loss.

This study has several limitations to be addressed. First, its cross-sectional nature makes it impossible to draw causal conclusions. Second, we defined interest diseases – specifically, PVD and cold injuries – based on ICD-10 diagnostic code previously used. However, the insurance claims database has an intrinsic limitation that has relevance to miscoding, misclassification, and misdiagnosis of any diseases. Thus, our data is also free from such criticism. Third, we focused on local injuries, but a broader classification of cold injuries was used when coded by the ICD-10. There exists a misclassification of local cold injuries. Further studies should analyze the detailed medical records on patient-reported symptoms, the affected body part, and diagnostic evaluations to clarify the risk of local cold injuries in PVD patients. Forth, in non-severe cold injuries, self-treatment at home could be made possible by the purchase of treatment supplies without visiting a hospital. It cannot be ruled out that the presence of cold injuries may have been relatively small and that this affected our observation. Finally, our data lacks important variables affecting cold injury risk. Variables include fatigue, inadequate clothing, and malnutrition [37, 38]. The association observed may be sensitive to unmeasured variables, and this should be considered.

## Conclusions

In conclusion, we found a significant association between PVD and cold-induced injuries in the general population. This association was predominant in PVD patients with low body fat involving underweight and normal weight patients. Our study suggests that the risk of cold induced injuries may be exacerbated by underlying conditions, specifically PVD and low body weight. With climate change waring more extreme weather events, weather-induced illnesses and injuries are of potential importance to human health. Given that cold injuries are as preventable as heat injuries, strategies to identify at-risk individuals and minimize health hazards are essential to cope with climate impacts.

## Data Availability

The data that support the findings of this study are available from the National Health Insurance Service in Korea but restrictions apply to the availability of these data, which were used under license for the current study, and so are not publicly available. Data are however available from the authors upon reasonable request and with permission of the National Health Insurance Service in Korea.

## Abbreviations

BMI: body mass index
CI: confidence interval
NHIS-NSC: National Health Insurance Service-National Sample Cohort
OR: Odds ratio
PVD: Peripheral vascular disease
